# Age, maturation and serum lipid parameters: findings from the German Health Survey for Children and Adolescents

**DOI:** 10.1186/s12889-019-7901-z

**Published:** 2019-12-03

**Authors:** Anja Schienkiewitz, Julia Truthmann, Andrea Ernert, Susanna Wiegand, Karl Otfried Schwab, Christa Scheidt-Nave

**Affiliations:** 1 Department of Epidemiology and Health Monitoring, Robert Koch-Institute, Berlin, Germany; 2 DZHK (German Centre for Cardiovascular Research), Berlin, Germany; 3 Institute for Biostatistics and Clinical Epidemiology, Charité Universitätsmedizin Berlin, Berlin, Germany; 4 Institute for Experimental Pediatric Endocrinology, Charité Universitätsmedizin Berlin, Berlin, Germany; 5 Department of Pediatrics and Adolescent Medicine, Faculty of Medicine, University of Freiburg, Freiburg im Breisgau, Germany

**Keywords:** Cholesterol, Lipoprotein, Population based study, Children, Adolescents

## Abstract

**Background:**

Recommendations on preventive lipid screening among children and adolescents remain controversial. The aim of the study was to assess age and puberty-related changes in serum lipids, including total cholesterol (TC), and high-density (HDL-C) and *non*-high-*density* lipoprotein cholesterol (Non-HDL-C).

**Methods:**

Using cross-sectional data from the National Health Interview and Examination Survey for Children and Adolescents in Germany (KiGGS 2003–2006; *N* = 13,676; 1–17 years), changes in distributions of serum lipids were visualized according to sex, age and maturation. Youth aged 10–17 years were classified as prepubescent, early/mid-puberty, and mature/advanced puberty. Multiple linear regressions were used to quantify the impact of pubertal stage on serum lipid levels, adjusted for potential confounding factors.

**Results:**

Among children 1–9 years mean serum lipid measures increased with age, with higher mean TC and Non-HDL-C among girls than boys. Among children 10–17 years, advanced pubertal stage was independently related to lower lipid measures. Adjusted mean TC, HDL-C and Non-HDL-C was 19.4, 5.9 and 13.6 mg/dL lower among mature/advanced puberty compared to prepubescent boys and 11.0, 4.0 and 7.0 mg/dL lower in mature/advanced puberty compared to prepubescent girls.

**Conclusions:**

Lipid concentrations undergo considerable and sex-specific changes during physical growth and sexual maturation and significantly differ between pubertal stages. Screening recommendations need to consider the fluctuations of serum lipids during growth and sexual maturation.

## Background

There is strong evidence that cardiovascular disease has its roots in childhood and that early lipoprotein abnormalities play a crucial role in the pathogenic process [1–3]. Recommendations on preventive lipid screening among children and adolescents remain controversial [4]. A high risk approach for all age groups is recommended by the American Academy of Pediatrics (AAP) [5] and the American Heart Association [6]. Universal screening of children 9–11 years is proposed by the Expert Panel on Integrated Guidelines for Cardiovascular Health and Risk Reduction in Children and Adolescents (National Heart, Lung, and Blood Institute; NHLBI) [7] and subsequently included into the AAP Bright Futures schedule for well-child supervision [8]. A systematic evidence review for the US Preventive Services Task Force found insufficient evidence to give any recommendations on screening and treatment for hypercholesterolemia in children and adolescents [9]. In Germany, the Working Group for pediatric metabolic disorders (APS) of the German Society for Pediatric and Adolescent Medicine (DGKJ) suggests a universal screening as part of the preventive check-up for children at the age of 5 years (U9 screening) [10]. Notably, children with familial hypercholesterinemia would benefit from early diagnosis [11]. However, no universal lipid screening has been implemented in Germany as of October 2019 [10, 12]*.*

There are a number of major unresolved issues about universal lipid screening in children [9, 13]. Current NHLBI integrated guidelines for cardiovascular health and risk reduction in children and adolescents do not sufficiently take into account physiological fluctuations in serum lipid concentrations during growth and maturation. Pubertal changes in serum lipids are considered by defining a specific age range for screening (9–11 years), which is assumed to precede puberty in the majority of children. During puberty hormonal changes associated with pubertal growth spurt and progressive maturation lead to marked increases of cholesterol requirement and consequently to decreases of lipid values [13]. Chronological age as well as sexual maturation are likely to be independent determinants of serum lipid levels in children and adolescents. Several cross-sectional [14–16] and longitudinal [17–19] population-based studies of children and adolescents have demonstrated that serum lipids increase with age until puberty and decline thereafter. Nevertheless, the interrelationship between serum lipid concentrations and physical growth remains poorly understood. Few studies have so far analyzed the distribution of serum lipids and lipoprotein levels according to chronological age as well as measures of pubertal status [18–21]. Moreover, the confounding effect of obesity, physical activity and dietary habits has not been well studied despite the association with lipid levels and considerable changes with age among children and adolescents [22, 23]. Previous studies in the US have shown that serum cholesterol levels vary according to ethnicity [24, 25].

Using data from a large nationally representative health survey of children and adolescents in Germany, we analyzed and visualized the independent contributions of chronological age and pubertal status on sex-specific distributions of serum lipids and lipoprotein levels among children and adolescents 1–17 years of age. We specifically asked whether findings were affected by age- and puberty-related changes in obesity, physical activity and dietary habits and also examined the interrelationship between immigration background, pubertal status and serum lipid concentrations.

## Methods

### Study design and study population

The German Health Interview and Examination Survey for Children and Adolescents (KiGGS) was conducted from May 2003 to May 2006 as a population-wide, nationally representative cross-sectional survey based on 17,640 participants aged 0 through 17 years (8985 boys and 8655 girls). The design, sampling strategy and study protocol have been previously described in detail [26]. In brief, based on a two-staged sampling procedure, first 167 study locations were selected proportional to the distribution of communities in Germany according to federal state, type of community, and population size. At the second stage within each sample point, children were sampled randomly from local population registries with stratification by sex and age. The overall response rate was 66.6%. We obtained written consent from parents of all participating children irrespective of the child’s age and additionally from participants 14 years of age and older. The study was approved by the Ethics Committee at the Charité Universitätsmedizin Berlin, Germany.

**Fig. 1:**
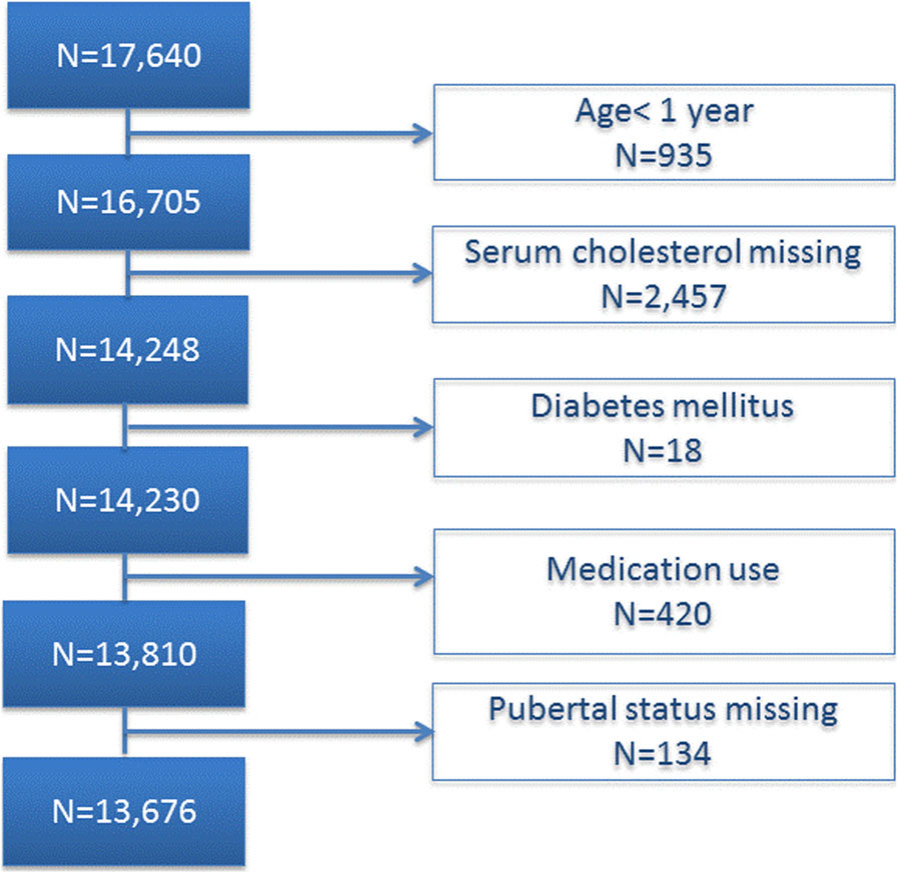
Flow diagram of study participants selection

For the present analysis, we excluded children under the age of 1 year (*n* = 935) and those with missing information on serum cholesterol measures (assessed among children 1 year of age and older; *n* = 2457). We also excluded participants with diabetes mellitus (*n* = 18) and those currently using lipid lowering drugs (*n* = 11), systemic corticosteroids (*n* = 18) or oral contraceptives (*n* = 391). Furthermore participants with missing information on pubertal status (assessed among children 10 years of age and older; *n* = 134) were excluded (Fig. 1). The final study population comprised 13,676 children and adolescents 1–17 years of age, 7187 boys and 6489 girls.

**Fig. 2:**
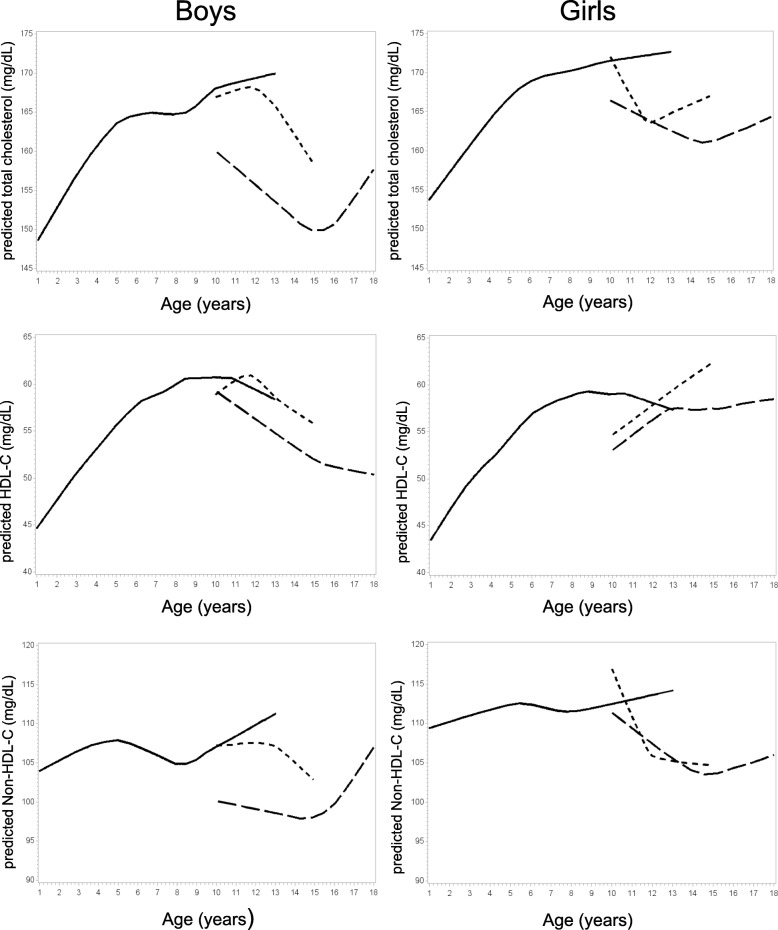
Distribution of serum lipid levels by sex and pubertal stage provided with selected smoothing parameters. HDL-C: high-density lipoprotein cholesterol; Non-HDL-C: non-high-density lipoprotein cholesterol; solid line = prepubertal stage, short broken line = pubertal stage, long broken line = postpubertal stage

### Data collection

Anthropometric measurements were performed by trained staff members based on standardized study procedures. Body height was measured to the nearest 0.1 cm using a portable Harpenden Stadiometer (Holtain Ltd., Crymych, UK). Body weight was measured wearing only underwear to the nearest 0.1 kg with a calibrated scale (SECA, Birmingham, UK) [26]. Body mass index (BMI) was calculated as weight in kilograms divided by the square of height in meters. Sex and age-specific BMI z-scores were calculated according to Schaffrath-Rosario et al. [27]. Obesity was defined using the International Obesity Task Force definition [28]. Information on physical activity was obtained based on self-report using standardized self-administered questionnaires for parents and older children and adolescents as previously described [26]. Information on current smoking was collected among children and adolescents 11 years of age and older based on self-report [29]. Participants were asked: “Do you currently smoke?” “daily“, „several times a week”,” once a week”,” more seldom” or “no”. To assess the physical activity level, boys and girls 11 years and older were asked how often they were physically active in their leisure time in a way they sweat or breathe hard: “every day”, “3 to 5 times a week”, “once to twice a week” or “never” [30]. Parents of children younger than 11 years rated the physical activity level of their child. Among 1–2 years of age the frequency of participation in swimming courses/children’s gymnastics was assessed as: “never”, “<1 time a week”, “≥1-2 times a week”, “every day”, and “several times a day”. Among 3–10 year-olds physical activity within and outside sports clubs was determined based on following categories: “never”, “1-2 times a month”, “1-2 times a week”, “3-5 times a week”, and “every day” [30]. For analysis physical activity level was classified into age-specific categories: low (1–2 years: never; 3–17 years: <1 time a week), middle (1–2 years: <1 time a week; 3–10 years: 1–2 times a week; 11–17 years: 3–5 times a week) and high (1–2 years: ≥1 time a week; 3–10 years: ≥3 times a week: 11–17 years: every day). To assess the usual intake of selected food groups a self-administered Food Frequency Questionnaire was filled in by children 11 years of age and older and parents of children younger than 11 years of age. The Healthy Food Diversity Index (HFD) was calculated based on 41 food items as described elsewhere [31]. The index considers three aspects: the number (n), distribution, and health value of all consumed foods and is bounded between 0 and 1–1/n. Higher HFD values reflect a healthier diet. Information on ethnicity was not collected in the KiGGS survey. Instead immigration background was defined based on self-reported information on the parents’ country of origin. Children were defined as having an immigration background if at least one of the parents was not born in Germany and/or had no German citizenship [32].

In this large epidemiological study self-assessed pubertal hair growth was used as proxy for pubertal status. Participants older than 10 years were asked to provide self-estimates of pubertal hair growth pattern assisted by standardized showcards displaying the six Tanner stages [33, 34]. Pubertal status was classified as prepubescent (Tanner stage 1), early/mid-puberty (Tanner stages 2–3), or mature/advanced puberty (Tanner stages 4–6) [35]. Pubertal stage was not assessed among children younger than 10 years. For the analysis children younger than 10 years were assumed to have prepubescent status.

Venous blood samples were taken at the study centers and the time of blood collection and hours since the last food intake were recorded. Blood specimens were processed within 1 h according to a highly standardized protocol [36, 37], serum aliquots were frozen and transported on frozen cool packs at − 50 °C to a central laboratory for analysis.

### Laboratory assays

Serum total (TC) and high density lipoprotein cholesterol (HDL-C) concentrations were analyzed in a central University Hospital Laboratory (German Cardiology Center, Virchow University Hospital, Charité, Berlin) using a fully enzymatic cholesterol oxidase PAP method and homogenous enzymatic color test (Roche, Mannheim, Germany) [37]. Non-HDL cholesterol (Non-HDL-C) was calculated as the difference between TC and HDL-C. Dyslipidemia was defined according to the Expert Panel on Integrated Guidelines for Cardiovascular Health and Risk Reduction in Children and Adolescents using the following cut-off values: TC ≥ 200 mg/dL, HDL-C ≥ 40 mg/dL and Non-HDL-C ≥ 145 mg/dL [7].

### Statistical analysis

Analyses were conducted using SAS release 9.4 (SAS Institute, Cary, NC). Tests were two-sided, and *p*-values < 0.05 were considered statistically significant. Prevalence estimates or means and 95%-confidence intervals (95%-CI) were calculated by sex and three age categories (1–9, 10–13, 14–17 years) for all study characteristics. The Cochran-Armitage test for trend was applied to test for group differences in categorical variables. Mean, median (P50), and 95th percentile (P95) and 95%-CI were calculated for TC and Non-HDL-C [7] by strata of sex, five age categories (1–2, 3–6, 7–9, 10–13, 14–17 years), and Tanner stage among children and adolescents ≥10 years of age. For HDL-C the 10th percentile was calculated [7].

Differences in demographic characteristics from the official German population according to age, gender, community size and residence were considered using survey specific weighting factors in order to adjust for the clustered sample design as well as non-response, as previously described in detail [26]. To take into account the weighting as well as the correlation of the participants within a community, the confidence intervals are determined with the survey procedures for complex samples of SAS 9.4.

For visualization of the age dependent development of the lipoproteins according to sexual maturation we performed locally weighted regression models (LOESS) and presented the predicted values graphically. As information on pre- and pubertal stage in children younger than 10 years of age was not obtained in KiGGS, some of the LOESS regression curves showed strong fluctuations due to individual data. Curves with higher smoothing parameters, which were chosen by visual inspection are presented in the manuscript. The original plots are provided in Additional file 1, along with a description of LOESS methods in Additional file 2.

Linear regression models were conducted to analyze the association between various lipid parameters (TC, HDL-C, Non-HDL-C) as dependent variables, and categories of pubertal status (prepubescent, early/mid-puberty, mature/advanced puberty) as the independent variable (Model 1). Model 2 was adjusted for chronological age by including a centered age-variable (subtracting the sample mean stratified for sex) and a centered and squared age-variable. Further adjustments were done for body mass index (z-score BMI; Model 3) and for behavioral determinants as smoking, nutrition and physical activity (Model 4). To estimate the proportion of variability estimated by the model the coefficient of determination of regression models (R squared) was used. Participants with missing values for any covariable were excluded from the regression analyses. All analyses were stratified for sex.

## Results

As shown in Tables 1, 21.7% of boys and 47.0% of girls 10–13 years of age classified as mature/advanced puberty, while the majority of boys (51.8%) and roughly one third (30.2%) of girls in this age group had early/mid-puberty status. In both sexes more than 90% of adolescents 14–17 years of age had mature/advanced puberty status. In both sexes, mean body weight, body height, and BMI as well as the prevalence of obese children increased with chronological age. The prevalence of highly physically active children and adolescents significantly declined with age and was consistently higher among boys than girls; this sex difference was most pronounced among adolescents 9–13 and 14–17 years of age. The HFD index declined with increasing age, most pronounced among boys. The proportion of children with immigration background did not vary by age group (Table 1).

**Table 1:** Characteristics of the study population (*n* = 13,676)

% or Mean (95%-CI)	N (missing)	1–9 years	10–13 years	14–17 years
Boys (n)		3529	1909	1749
Pubertal Stage^a^	–			
Prepubescent		n.a.	26.5 (24.2–28.9)	0.1 (0.0–0.4)
Early/mid-puberty		n.a.	51.8 (49.2–54.3)	5.7 (4.6–7.0)
Mature/advanced puberty		n.a.	21.7 (19.6–24.0)	94.3 (92.9–95.4)
Dyslipidemia^b^	**–**			
TC ≥ 200 [mg/dL]		8.0 (7.0–9.1)	10.2 (8.6–12.0)	4.8 (3.8–5.9)
HDL-C < 40 [mg/dL]		10.9 (9.6–12.3)	6.3 (5.2–7.7)	15.1 (13.2–17.3)
Non-HDL-C ≥ 145 [mg/dL]		7.2 (6.2–8.3)	8.8 (7.4–10.5)	6.1 (5.1–7.4)
Anthropometry				
Height (cm)	16	114.5 (113.9–115.1)	153.3 (152.7–153.8)	175.4 (174.9–175.8)
Weight (kg)	23	22.2 (21.9–22.4)	45.8 (45.1–46.5)	67.4 (66.6–68.1)
Body Mass Index (kg/m^2^)	34	16.3 (16.3–16.4)	19.2 (19.0–19.4)	21.8 (21.6–22.0)
Obesity^c^	24 (582)	3.4 (2.9–4.1)	4.7 (3.7–6.0)	6.5 (5.4–7.8)
Physical Activity/week	334			
Low		26.8 (24.9–28.8)	25.2 (22.8–27.8)	38.1 (35.6–40.6)
Middle		32.6 (30.6–34.6)	36.2 (33.4–39.0)	36.9 (34.7–39.2)
High		40.6 (38.6–42.7)	38.6 (35.8–41.6)	25.0 (23.0–27.2)
Healthy Food Diversity Index	327	0.55 (0.54–0.56)	0.52 (0.51–0.52)	0.48 (0.47–0.49)
Smoking	40	n.a.	3.9 (2.8–5.3)	30.8 (28.4–33.3)
Immigration background	23	16.5 (14.3–19.0)	18.8 (16.1–21.9)	17.0 (14.5–19.7)
Girls (n)		3384	1805	1300
Pubertal Stage^a^	–			
Prepubescent		n.a.	22.7 (20.5–25.1)	–
Early/mid-puberty		n.a.	30.2 (27.9–32.7)	3.5 (2.6–4.7)
Mature/advanced puberty		n.a.	47.0 (44.6–49.4)	96.5 (95.3–97.4)
Dyslipidemia^b^	**–**			
TC ≥ 200 [mg/dL]		10.7 (9.5–12.1)	11.0 (9.6–12.5)	9.6 (7.8–11.7)
HDL-C < 40 [mg/dL]		12.8 (11.5–14.3)	7.5 (6.2–9.1)	5.6 (4.2–7.4)
Non-HDL-C ≥ 145 [mg/dL]		10.4 (9.1–11.8)	8.7 (7.3–10.2)	6.8 (5.2–8.8)
Anthropometry				
Height (cm)	6	113.5 (113.0–114.1)	153.7 (153.3–154.2)	164.9 (164.5–165.3)
Weight (kg)	25	21.6 (21.4–21.8)	46.8 (46.1–47.5)	59.7 (58.9–60.5)
Body Mass Index (kg/m^2^)	30	16.2 (16.1–16.2)	19.6 (19.3–19.8)	21.9 (21.7–22.2)
Obesity^c^	26 (577)	3.5 (2.7–4.4)	4.7 (3.8–6.0)	5.5 (4.2–7.1)
Physical Activity/week	332			
Low		26.6 (24.6–28.8)	40.5 (38.1–43.0)	61.2 (58.0–64.3)
Middle		36.9 (34.8–38.9)	31.9 (29.6–34.3)	25.4 (22.5–28.5)
High		36.5 (34.3–38.8)	27.6 (25.2–30.1)	13.4 (11.3–15.7)
Healthy Food Diversity Index	308	0.56 (0.55–0.56)	0.55 (0.54–0.56)	0.54 (0.53–0.55)
Smoking	20	n.a.	3.2 (2.3–4.4)	24.6 (22.0–27.3)
Immigration background	36	16.3 (14.2–18.7)	18.1 (15.5–21.1)	19.1 (16.0–22.5)

*n.a.* not assessed

^a^The stage of puberty defined according to Tanner stage was only collected for boys and girls aged 10 years and older: prepubescent = stage 1, early/mid-puberty = stage 2–3, and mature/advanced puberty = stage 4–6

^b^according the Expert Panel on Integrated Guidelines for Cardiovascular Health and Risk Reduction in Children and Adolescents [7]

^c^Obesity defined according to International Obesity Task Force [28]; not assessed at the age of 1–2 years (boys: *N* = 558, girls: *N* = 551)

The prevalence of children meeting criteria for dyslipidemia according to guideline-based cutoff values varied by sex and age categories. Among boys, the prevalence of dyslipidemia based on TC cutoffs among boys 14–17 years of age was significantly lower compared to boys 10–13 years and 1–9 years of age (4.8% vs. 10.2% and 80%). Similar albeit less pronounced differences were observed for Non-HDL-C, whereas the prevalence of low-HDL-C showed substantial fluctuations across age categories from 10.9% among boys 1–9 years of age to 6.3% among those 10–13 years of age to 15.1% among boys 14–17 years of age (Table 1). Among girls, changes in the prevalence of dyslipidemia across age categories were less pronounced except for a substantially decreasing prevalence in low HDL-C ranging from 12.8% in the youngest to 5.6% in the oldest age group. While the prevalence of dyslipidemia did not differ between sexes among children 10–13 years of age, girls were significantly more likely than boys to exceed cutoff criteria for TC and Non-HDL-C in the youngest age group (Table 1). Among children 14–17 years of age, girls were also significantly more likely than boys to exceed TC cutoff criteria (9.6% vs. 4.8%) and significantly less likely than boys to exceed HDL-C criteria (5.6% vs. 15.1%).

In both sexes, mean serum lipids generally increased with age until age group 7–9 (until 3–6 years for Non-HDL-C) and decreased thereafter (Tables 2 and 3). Among children and adolescents 10 years of age and older lipid concentrations, except HDL-C among girls, consistently decreased with increasing Tanner stage. Among boys, a distinct decline in mean serum lipid levels was observed between Tanner stages 3 and 4, whereas a gradual decline in mean lipid concentrations with increasing Tanner stages was evident among girls. Figure 2 presents the distribution of mean serum lipid values according to sex, chronological age and pubertal status. Beyond age 10 years, boys and girls at prepubescent stages had considerably higher mean TC concentrations compared to their peers at early/mid-puberty or mature/advanced puberty status independent of chronological age. In both sexes, the nadir of mean TC was observed among mature/advanced puberty at 15 years of age (148 mg/dL among boys vs. 159 mg/dL among girls). Beyond this age, mean TC was observed to rise again. Similar patterns were observed for mean Non-HDL-C. The nadir for mean Non-HDL-C observed at age 15 years amounted to 97 mg/dL among boys vs. 102 mg/dL among girls. Mean TC was consistently higher among girls than boys across all age groups and categories of pubertal status. The age-related increase in Non-HDL-C beyond age 15 years was considerably steeper among boys than girls. Differences between mature/advanced puberty vs. prepubescent children were larger among boys than girls. Boys 10 years and older with advanced pubertal stage had significantly lower mean HDL-C compared to those of prepubescent status. Among mature/advanced puberty boys, mean HDL-C levels further decreased, whereas mean HDL-C values did not substantially change with age among mature/advanced puberty girls.

**Table 2:** Serum lipid levels in boys by age group and Tanner stage (≥ 10 years)

		N	TC [mg/dL]	HDL-C [mg/dL]	Non-HDL-C [mg/dL]
Age group		7187			
1–2	Mean	558	153.0 (150.0–156.0)	47.9 (46.8–49.1)	105.1(102.0–108.1)
	P50		151.1 (148.4–153.9)	48.0 (46.9–49.8)	102.7 (99.6–105.9)
	P95/P10^a^		196.3 (189.8–202.7)	32.8 (30.5–35.0)	151.1 (141.9–160.3)
3–6	Mean	1544	163.3 (161.4–165.2)	55.6 (54.8–56.4)	107.7 (105.8–109.6)
	P50		161.2 (159.2–163.2)	54.5 (53.5–55.5)	105.2 (103.3–107.2)
	P95/P10 ^a^		212.6 (206.5–218.7)	40.4 (38.9–41.8)	153.9 (149.1–158.6)
7–9	Mean	1427	165.2 (163.6–166.7)	60.0 (59.1–60.9)	105.1 (103.5–106.7)
	P50		162.9 (160.7–165.1)	59.5 (58.8–60.5)	103.0 (101.0–105.0)
	P95/P10 ^a^		208.3 (205.8–210.9)	43.7 (42.4–45.1)	146.0 (143.1–148.9)
10–13	Mean	1909	164.5 (162.8–166.2)	59.0 (58.2–59.7)	105.6 (103.9–107.2)
	P50		161.8 (160.1–163.5)	57.9 (56.9–58.9)	102.2 (100.3–104.1)
	P95/P10 ^a^		214.2 (209.7–218.8)	42.5 (41.5–43.5)	155.1 (151.8–158.4)
14–17	Mean	1749	152.3 (150.8–153.9)	51.4 (50.8–52.1)	100.9 (99.3–102.5)
	P50		149.6 (147.8–151.3)	50.1 (49.2–51.0)	97.2 (95.6–98.9)
	P95/P10 ^a^		198.2 (195.0–201.5)	37.9 (37.2–38.6)	149.1 (145.0–153.1)
Tanner stage (≥10 years)^b^		3658			
1	Mean	509	169.2 (166.1–172.3)	60.6 (59.4–61.9)	108.5 (105.6–111.5)
	P50		166.9 (163.3–170.4)	60.5 (58.8–62.2)	105.3 (101.2–109.4)
	P95/P10 ^a^		213.1 (104.1–222.1)	43.3 (40.9–45.6)	154.2 (149.4–158.9)
2	Mean	727	167.5 (165.3–169.7)	59.7 (58.6–60.8)	107.8 (105.5–110.1)
	P50		164.9 (162.1–167.7	58.9 (57.3–60.4)	104.4 (101.1–107.7)
	P95/P10 ^a^		215.9 (210.2 (221.7)	43.3 (42.0–44.6)	158.9 (153.6–164.1)
3	Mean	374	164.0 (160.5–167.5)	58.7 (57.1–60.4)	105.2 (101.7–108.8)
	P50		161.0 (156.9–165.0)	57.0 (55.3–58.7)	101.7 (97.0–106.4)
	P95/P10 ^a^		217.1 (206.7–227.6)	42.7 (40.7–44.8)	156.4 (143.0–169.6)
4	Mean	458	154.1 (151.5–156.7)	54.0 (52.5–55.5)	100.2 (97.5–102.8)
	P50		153.1 (150.0–156.2)	52.3 (50.5–54.1)	96.6 (93.8–99.5)
	P95/P10 ^a^		198.9 (192.7–205.1)	38.7 (36.8–40.6)	145.3 (139.9–150.7)
5	Mean	1069	150.0 (148.0–151.9)	51.9 (51.1–52.6)	98.1 (96.1–100.1)
	P50		147.4 (144.8–150.0)	50.4 (49.4–51.4)	95.3 (93.5–97.2)
	P95/P10 ^a^		193.8 (189.0–198.5)	38.1 (37.2–38.9)	140.6 (124.0–147.3)
6	Mean	521	154.2 (151.5–157.0)	50.0 (49.0–51.0)	104.2 (101.5–106.9)
	P50		151.3 (147.3–155.2)	49.1 (47.9–50.4)	100.9(98.3–103.6)
	P95/P10 ^a^		202.9 (196.0–209.8)	37.2 (35.7–38.7)	156.5 (148.9–164.0)

^a^P95 for TC and non-HDL-C and P10 for HDL-C according to the cut-offs for high plasma lipid concentrations for children and adolescents from the Expert Panel on Integrated Guidelines for Cardiovascular Health and Risk Reduction in Children and Adolescents [7]

^b^The stage of puberty defined according to Tanner stage was only collected for boys and girls aged 10 years and older

**Table 3:** Serum lipid levels in girls by age group and Tanner stage (≥ 10 years)

		n	TC [mg/dLl]	HDL-C [mg/dL]	Non-HDL-C [mg/dL]
Age group		6489			
1–2	Mean	551	157.2 (154.4–160.0)	47.5 (46.3–48.8)	109.7 (107.0–112.4)
	P50		155.9 (153.1–158.7)	46.8 (45.4–48.2)	107.5 (104.5–110.5)
	P95/P10^a^		202.6 (194.2–211.1)	32.5 (30.1–34.9)	152.1 (148.3–155.8)
3–6	Mean	1484	166.8 (165.0–168.6)	54.4 (53.6–55.2)	112.4 (110.6–114.2)
	P50		164.9 (163.0–166.7)	54.0 (53.2–54.8)	105.2 (103.3–107.2)
	P95/P10 ^a^		212.6 (209.7–215.6)	38.7 (37.4–40.0)	153.9 (149.1–158.6)
7–9	Mean	1349	170.8 (169.1–172.5)	59.2 (58.4–59.9)	111.6 (110.0–113.3)
	P50		168.8 (167.3–170.4)	58.3 (57.4–59.2)	110.3 (108.5–112.1)
	P95/P10 ^a^		215.4 (210.8–219.9)	44.0 (42.8–45.2)	157.4 (151.3–163.5)
10–13	Mean	1805	165.5 (164.0–166.9)	57.4 (56.7–58.1)	108.1 (106.7–109.6)
	P50		163.1 (161.5–164.6)	56.4 (55.5–57.3)	105.6 (104.1–107.2)
	P95/P10 ^a^		212.8 (207.9–217.6)	41.8 (40.8–42.8)	157.4 (152.7–162.1)
14–17	Mean	1300	162.1 (160.2–163.9)	57.9 (57.1–58.8)	104.1 (102.1–106.1)
	P50		159.1 (157.3–161.0)	57.1 (56.2–58.1)	101.2 (98.7–103.7)
	P95/P10 ^a^		211.7 (206.9–216.5)	42.8 (41.7–44.0)	151.7 (145.9–157.4)
Tanner stage (≥10 years)^b^		3105			
1	Mean	424	171.5 (168.7–174.4)	58.4 (57.0–59.8)	113.1 (110.3–116.0)
	P50		168.2 (165.4–170.9)	57.7 (56.2–59.3)	109.0 (106.3–111.7)
	P95/P10 ^a^		219.7 (210.2–229.3)	41.4 (38.9–43.9)	164.2 (156.5–171.8)
2	Mean	336	167.0 (163.5–170.5)	57.7 (55.9–59.4)	109.3 (105.6–113.0)
	P50		163.4 (159.5–166.9)	56.2 (54.1–58.3)	108.2 (104.1–112.2)
	P95/P10 ^a^		209.5 (201.0–218.0)	41.5 (37.8–45.2)	151.0 (141.7–160.3)
3	Mean	258	163.7 (159.9–167.6)	57.7 (56.0–59.4)	106.1 (101.9–110.3)
	P50		162.9 (158.0–167.9)	57.1 (55.3–58.9)	106.2 (99.7–112.7)
	P95/P10 ^a^		208.8 (201.7–215.8)	42.3 (38.6–46.1)	146.1 (138.1–154.1)
4	Mean	685	163.9 (161.3–166.5)	57.5 (56.4–58.7)	106.4 (103.9–108.9)
	P50		160.3 (157.4–163.3)	57.1 (55.8–58.3)	103.6 (100.9–106.3)
	P95/P10 ^a^		209.8 (203.5–216.2)	42.3 (40.7–43.8)	157.5 (149.3–165.6)
5	Mean	1110	161.5 (159.6–163.4)	57.6 (56.7–58.5)	103.9 (101.9–105.9)
	P50		158.8 (156.7–16,094)	56.3 (55.3–57.3)	100.9 (98.7–103.0)
	P95/P10 ^a^		209.5 (203.0–216.0)	42.5 (41.3–43.7)	151.3 (143.6–159.0)
6	Mean	292	160.4 (156.0–164.8)	57.0 (55.4–58.6)	103.4 (99.2–107.7)
	P50		155.8 (150.9–160.7)	56.3 (54.2–58.3)	98.9 (94.7–103.2)
	P95/P10 ^a^		217.1 (204.0–230.2)	42.9 (39.7–46.1)	156.2 (141.8–170.5)

^a^P95 for TC and non-HDL-C and P10 for HDL-C according to the cut-offs for high plasma lipid concentrations for children and adolescents from the Expert Panel on Integrated Guidelines for Cardiovascular Health and Risk Reduction in Children and Adolescents [7]

^b^The stage of puberty defined according to Tanner stage was only collected for boys and girls aged 10 years and older

Among both sexes mature/advanced pubertal status showed an inverse association with TC, HDL-C and Non-HDL-C (Tables 4 and 5). Among girls, early/mid-puberty status was associated with an increased HDL-C level. After adjusting for chronological age pubertal status showed an inverse association with TC, HDL-C and Non-HDL-C among both sexes. Results persisted after additional adjustment for BMI z-score (Model 3). Further adjustment for behavioral determinants did not materially change the results (Model 4). The difference in mean adjusted serum lipid concentrations with advanced pubertal stage remained substantial: the adjusted mean TC was 19.4 mg/dL lower in mature/advanced puberty compared to prepubescent boys and 10.9 mg/dL lower in mature/advanced puberty compared to prepubescent girls. Values for explained variance for the association between pubertal status and different cholesterol parameters adjusted for anthropometric variables were very small except for HDL-C and consistently somewhat higher among boys compared to girls (Tables 4 and 5). BMI z-score was significantly associated with serum cholesterol parameters (Model 3 and 4). The behavioral determinants showed no independent associations with TC, HDL-C or non-HDL-C, except for smoking which was inversely associated with HDL-C among girls (Model 4). Linear regression analyses were repeated adding immigration background to the models including pubertal status and chronological age (Model 2). Immigration background did not independently contribute to this model and results regarding the association of pubertal status with serum lipids persisted (Additional file 3). There was also no evidence for first order interaction, e. g. modification of the association between pubertal status and serum lipids by immigration background.

**Table 4:** Linear Regression of serum cholesterol parameters on pubertal stage among boys adjusted for age and behavioral determinants (*n* = 6531)

		TC [mg/dL]	HDL-C [mg/dL]	Non-HDL-C [mg/dL]
Model 1: unadjusted	R^2^	*0.04*	*0.04*	*0.01*
Early/mid-puberty	β (S.E.)	*2.7 (1.2)* ^*^	*3.0 (0.5)* ^***^	*−0.3 (1.1)*
Mature/advanced puberty	β (S.E.)	*−10.8 (0.9)* ^***^	*−4.5 (0.4)* ^***^	*−6.3 (0.9)* ^***^
Model 2: adj. For age	R^2^	*0.05*	*0.11*	*0.01*
Early/mid-puberty	β (S.E.)	*−4.3 (1.4)* ^*^	*−1.9 (0.7)* ^*^	*−2.4 (1.4)*
Mature/advanced puberty	β (S.E.)	*−18.7 (1.8)* ^***^	*−6.8 (1.0)* ^***^	*−11.8 (1.8)* ^***^
Age	β (S.E.)	0.2 (0.3)	−0.9 (0.2)^***^	1.2 (0.4)^*^
Age (squared)	β (S.E.)	−0.1 (0.0)^*^	− 0.1 (0.0)^***^	0.1 (0.0)^*^
Model 3: adj. For age, BMI	R^2^	*0.06*	*0.16*	*0.05*
Early/mid-puberty	β (S.E.)	*−4.5 (1.4)* ^*^	*−1.6 (0.7)* ^*^	*−2.9 (1.4)* ^*^
Mature/advanced puberty	β (S.E.)	*−19.5 (1.8)* ^***^	*−5.9 (1.0)* ^***^	*−13.6 (1.7)* ^***^
Age	β (S.E.)	0.3 (0.3)	−1.1 (0.2)^***^	1.4 (0.3)^***^
Age (squared)	β (S.E.)	−0.1 (0.0)^*^	− 0.2 (0.0)^***^	0.1 (0.0)^*^
BMI	β (S.E.)	2.5 (0.4)^***^	−2.9 (0.2)^***^	5.4 (0.4)^***^
Model 4: adj. For age, BMI and behavioral determinants	R^2^	*0.06*	*0.16*	*0.05*
Early/mid-puberty	β (S.E.)	−4.4 (1.4)^*^	*−1.6 (0.7)* ^*^	*−2.8 (1.4)*
Mature/advanced puberty	β (S.E.)	*−19.4 (1.8)* ^***^	*−5.9 (1.0)* ^***^	*−13.6 (1.8)* ^***^
Age	β (S.E.)	0.2 (0.4)	−1.1 (0.2)^***^	1.2 (0.4)^*^
Age (squared)	β (S.E.)	−0.1 (0.0)^*^	−0.2 (0.0)^***^	0.1 (0.0)^*^
BMI	β (S.E.)	2.4 (0.4)^***^	−2.9 (0.2)^***^	5.3 (0.4)^***^
Smoking (yes vs. no)	β (S.E.)	1.5 (1.6)	−0.8 (0.6)	2.3 (1.6)
Healthy food diversity index	β (S.E.)	1.4 (2.5)	−0.6 (1.2)	2.0 (2.6)
Low physical activity	β (S.E.)	1.0 (1.0)	−0.5 (0.4)	1.5 (0.9)
Middle physical activity	β (S.E.)	0.3 (1.0)	−0.4 (0.4)	0.7 (0.9)

Model 2: adjusted for age (centred and squared)

Model 3: adjusted for age (centred and squared) and BMI (kg/m^2^, z-score)

Model 4: adjusted for age (centred and squared), BMI (kg/m^2^, z-score), smoking, healthy food diversity index and physical activity

*β* Regression coefficient, *S.E.* Standard error, *R*^*2*^ proportion of variance explained by the model, Reference = prepubescent stage

^*^p < 0.05, ^**^p < 0.001, ^***^p < 0.0001

**Table 5:** Linear Regression of serum cholesterol parameters on pubertal stage among girls adjusted for age and behavioral determinants (*n* = 5878)

		TC [mg/dL]	HDL-C [mg/dL]	Non-HDL-C [mg/dL]
Model 1: unadjusted	R^2^	*0.01*	*< 0.01*>	*0.01*
Early/mid-puberty	β (S.E.)	*−1.3 (1.5)*	*−2.3(0.7)* ^**^	*−3.6 (1.5)*
Mature/advanced puberty	β (S.E.)	*−4.6 (0.9)* ^***^	*2.5 (0.4)* ^***^	*−7.1 (0.9)* ^***^
Model 2: adj. For age	R^2^	*0.02*	*0.08*	*0.02*
Early/mid-puberty	β (S.E.)	*−7.3 (1.7)* ^***^	*−4.5 (0.8)* ^***^	*−2.8 (1.8)*
Mature/advanced puberty	β (S.E.)	*−10.2 (2.0)* ^***^	*−4.9 (0.8)* ^***^	*−5.2 (1.9)* ^*^
Age	β (S.E.)	−0.3 (0.4)	0.2 (0.2)	−2.8 (1.9)
Age (squared)	β (S.E.)	−0.1 (0.0)^***^	−0.1 (0.0)^***^	−5.2 (1.9)
Model 3: adj. For age, BMI	R^2^	*0.02*	*0.11*	*0.03*
Early/mid-puberty	β (S.E.)	*−7.4 (1.7)* ^***^	*−4.3 (0.8)* ^***^	*−3.2 (1.8)*
Mature/advanced puberty	β (S.E.)	*−10.7 (2.0)* ^***^	*−3.9 (0.8)* ^***^	*−6.8 (1.9)* ^**^
Age	β (S.E.)	−0.2 (0.4)	0.0 (0.2)	−0.2 (0.4)
Age (squared)	β (S.E.)	−0.1 (0.0)	−0.1 (0.0)^***^	0.0 (0.0)
BMI	β (S.E.)	1.2 (0.5)^*^	−2.2 (0.2)^***^	3.4 (0.5)^***^
Model 4: adj. For age, BMI and behavioral determinants	R^2^	*0.02*	*0.11*	*0.03*
Early/mid-puberty	β (S.E.)	*−7.6 (1.7)* ^***^	*−4.4 (0.8)* ^***^	*−3.2 (1.8)*
Mature/advanced puberty	β (S.E.)	*−10.9 (2.0)* ^***^	*−3.9 (0.8)* ^***^	*−7.0 (2.0)* ^**^
Age	β (S.E.)	−0.1 (0.4)	0.0 (0.2)	−0.2 (0.4)
Age (squared)	β (S.E.)	−0.1 (0.0)^**^	−0.1 (0.0)	0.0 (0.0)
BMI	β (S.E.)	1.2 (0.5)^*^	−2.2 (0.2)^***^	3.4 (0.5)^***^
Smoking (yes vs. no)	β (S.E.)	−2.9 (2.0)	−2.3 (0.9)^*^	−0.6 (2.1)
Healthy food diversity index	β (S.E.)	5.2 (2.8)	1.2 (1.5)	4.0 (2.6)
Low physical activity	β (S.E.)	1.0 (1.0)	−0.4 (0.5)	1.4 (1.0)
Middle physical activity	β (S.E.)	1.1 (1.0)	−0.5 (0.5)	1.6 (1.0)

Model 2: adjusted for age (centred and squared)

Model 3: adjusted for age (centred and squared) and BMI (kg/m^2^, z-score)

Model 4: adjusted for age (centred and squared), BMI (kg/m^2^, z-score), smoking, healthy food diversity index and physical activity

*β* Regression coefficient, *S.E.* Standard error, *R*^*2*^ proportion of variance explained by the model, Reference = prepubescent stage

^*^p < 0.05, ^**^p < 0.001, ^***^p < 0.0001

## Discussion

In this large cross-sectional study, which was representative of German children and adolescents 1 through 17 years, we examined and visualized the distribution of serum lipoprotein concentrations according to sex, chronological age, and different stages of sexual maturation. The results of the present study add strong evidence to previous observations that lipid concentrations during puberty are highly variable and significantly differ on average from lipid concentrations observed during prepubescent and mature/advanced puberty stages. Furthermore, the results were not explained by considerable age-related changes in body mass index or behavioral determinants of serum lipids, such as current smoking, food diversity and physical activity. As previously shown [18, 20], our results also emphasize pronounced sex differences with regard to mean lipid concentrations and fluctuations during natural growth and maturation. Consequently, the prevalence of dyslipidemia as defined by uniform NHLBI cut off values varied considerably by age and sex, which underlines the need for population-based reference data stratified by age, sex and pubertal status.

During the pubertal growth spurt cholesterol is included into the growing cells leading to decreases of lipid values. The pubertal growth spurt among girls is characterized by increased estrogen and progesterone levels and by increased testosterone levels among boys [19, 38]. It starts soon after the onset of puberty among girls and somewhat later among boys [39]. The considerable sex-specific differences in serum lipids in relation to sexual maturation and pubertal growth spurt of girls and of boys lead to more pronounced decreases of lipid values in boys compared to girls, because male pubertal growth spurt is more marked compared to that of girls [40].

While a representative sample of healthy French children aged 7–20 years indicated only little variation of TC and HDL-C according to age and gender [41], several other previous studies based on age-specific analyses demonstrated considerable variation according to chronological age [18, 20]. Furthermore, data of the present study indicate substantial variation in serum lipid concentration according to sexual maturation, independent of chronological age. Among boys, a steep decline in mean serum lipid levels consistently occurred between Tanner stage 3 and 4, whereas among girls mean serum lipids except HDL-C gradually declined between all Tanner stages. These patterns are likely to reflect sex-specific differences in pubertal growth spurt and are in accordance with results from a number of previous studies considering chronological age as well as pubertal maturation as potential determinants of serum lipid levels. Bertrais et al. [20] reported that prepubescent children have higher mean levels of TC and Triglycerides than those at mature/advanced puberty stage, but data for HDL-C and Non-HDL-C was not collected in this previous study [42]. Eissa et al. [18] also reported decreasing levels of TC and non-HDL-C during puberty with differences according to sex and race. Our finding of increasing TC values for boys and girls of 16 years and above is supported by longitudinal data from the Amsterdam Health and Growth Study [17] and two nationwide cross-sectional US studies [14, 15]. Similarly, our finding of a considerably steeper pubertal decline in HDL-C among boys than girls is in agreement with previous population-based longitudinal studies of US children and adolescents [18, 19].

Compared to prepubescent status higher HDL-C levels were found in early-mid-puberty. This reflects the strong increase in HDL-C levels with increasing chronological age as well as the fact that all children younger than 10 years were categorized as prepubescent. Adjusting for chronological age the effect for pubertal status reversed reflecting the rise in HDL-C in early puberty and the decline in HDL-C among girls and boys with mature or advanced pubertal status. Further adjustment for anthropometric indicators slightly reduced the inverse association between HDL-C levels and pubertal stage in our study. This is not surprising, because an age- and sex-specific measure of BMI (z-scores) was used, and the prevalence of relative obesity significantly increased with age. We have previously shown in KiGGS that overweight and obesity are significantly and positively associated to lipid measures and other cardiovascular risk factors among adolescent boys and girls independent of age and pubertal stage [22]. Increased duration of physical activity [43] as well as a healthy diet including whole grains, fish, fruits and vegetables [44] decrease TC levels. In our study physical activity and healthy food diversity showed no independent association with serum lipids in linear regression analyses also adjusting for chronological age and BMI. This may partly result from the complex interrelationship with BMI which cannot be disentangled in this cross-sectional study. We also cannot rule out that misclassification for behavioral determinants contributed to underestimate the association with serum lipids in the present study.

Our study has several limitations. First, KiGGS is a cross-sectional survey and the results of the present study preclude any conclusions on causality. Only longitudinal data could give answers on tracking of individual serum lipid levels during puberty. Second, the presented KiGGS data based on non-fasting lipoprotein measurements. Data from the US nationally cross-sectional NHANES Survey 1999–2008 available for 12,774 children aged 3–17 years indicated that those who had fasted and those who had not fasted before a lipid screening test show only small differences, which are probably not clinically important [45]. In a cohort with type 1 diabetes patients aged 1–20 years fasting had no relevant influence on TC and HDL-C [46]. Therefore, we assume that fasting measurements would not change our results. Third, we cannot exclude misclassification of pubertal stage due to self-assessment. Moreover, pubic hair status was used as proxy for Tanner stages, and breast/genital stages as well as additional measures as bone age or orchiometry were not obtained. Girls tend to underestimate their puberty stage by Tanner drawings and boys tend to overestimate their stage of development [47]. Nevertheless, self-assessment of pubertal status is an important time- and cost-saving tool in study settings where direct examinations are not feasible [48]. Fourth, the ascertainment of pubertal stage might be incomplete, as information on pubertal hair development was collected only among children and adolescents 10 years and older, with nearly 50% of girls 10–13 years showing an advanced pubertal stage. Secular trends in pubertal acceleration have been well documented in US and European girls [49] and boys [50], and the duration of puberty has been prolonged [51]. Thus, we may have underestimated the proportion of children already undergoing puberty, especially among girls. Fifth, there were limitations to the assessment of immigration background and behavioral determinants which were considered as covariables in the present analysis. Considerable heterogeneity of the group of children with immigration background may have masked an association with serum lipids in the present study. The definition of immigration background entirely relied on self-reported information on the parents’ country of origin. In lack of any specific information on ethnic background, further stratification of analyses according to subgroups of children with immigration background was not possible. Physical activity, dietary habits and smoking were assessed with self-administered questionnaires involving the potential for misclassification bias. Thus, the association of behavioral determinants with serum lipids might have been underestimated.

In the present study all R squared values were quite low. The low estimates among girls may partly reflect misclassification for pubertal status and behavioral covariables as described above in the limitations section. Nevertheless, the aim of the regression analysis was not to examine the explained variance in the model but rather to observe if there was a change in the association between pubertal stage and serum lipid levels under consideration of anthropometric markers or behavioral factors.

Despite these limitations the results of the present study are based on a large nationally representative sample of 1–17 year old children and adolescents in Germany and a selection bias can be neglected [26]. Furthermore, the distribution of TC- and Non-HDL-C-levels in children and adolescents across pubertal stages (prepubescent, early/mid-puberty, mature/advanced puberty) generally corresponded with previously published cross-sectional [21, 52] and longitudinal studies [18, 19]. Thus, the results are generalizable to western children and adolescents from Europe and North America. Considering pubertal stage in the assessment of lipid profiles in children and adolescents has practical implications. A study from the US show that TC levels in childhood explain 25–50% of the variability of values in adulthood [53]. Moreover, it is well known that tracking of unfavorable lipid and lipoprotein concentrations through life can induce processes of atherosclerotic cardiovascular changes [2, 3]. Unfavorable lipid values often result in medical treatment by dietary interventions and pharmacotherapy recommended by national guidelines [7]. The current US clinical guideline recommends comprehensive lipid screening for age group 9–11 years as a stable time for lipid assessment in children, based on the rationale that this time point will precede the onset of puberty for most children. Among 10–13 year old children in KiGGS, 47% of girls and 26.5% boys were postpubertal and 30.2% of girls and 51.8% of boys were pubertal. In addition, results from the Bogalusa Heart Study indicated that children with abnormal lipid values may show a substantially decrease in lipid values in the absence of any intervention [54]. The present results do not support current recommendations for routine screening among 9–11 year olds as the time of puberty is not suited for preventive serum screening. In Germany, universal screening at the age of 5 years has been suggested by the Working Group for pediatric metabolic disorders (APS) of the German Society for Pediatric and Adolescent Medicine (DGKJ) [10]. In KiGGS, 8% of five-year-old boys and 14% of five-year-old girls exceeded the NHLBI cut-off for TC. Despite the fact that the age of 5 years precedes the onset of puberty‚ the usefulness of uniform cut-off values for the definition of dyslipidemia remains unclear. Longitudinal studies are necessary to investigate the complex association between pubertal stage, pubertal growth spurt and serum lipids in childhood and adolescence and tracking of lipid levels among specific subgroups into adulthood. In addition, NHLIB guideline cut-off values are based on population-based reference data derived about 20 years ago. Population-based studies are needed to derive updated serum lipid and lipoprotein distributions among children and adolescents according to sex, chronological age and pubertal status. These studies should be periodically repeated in order to detect and explain changes over time. A recent study of trends in serum lipid and lipoprotein concentrations among US youths 6–19 years of age found favorable changes over time, which were consistent within subgroups of age, sex and ethnicity. The authors of this previous study pointed out that it would have been important to also stratify by pubertal status, however this information was not available [55].

## Conclusions

Results from this large nationwide study add to existing evidence that circulating lipids and lipoproteins undergo considerable and sex-specific changes during physical growth and sexual maturation. Changes in TC, HDL-C and non-HDL-C persisted after adjusting for behavioral determinants. A general lipid screening among children 9–11 years of age cannot be supported. Longitudinal studies are needed to provide further evidence on factors related to tracking of lipid levels from childhood and adolescence into young adulthood. Besides, periodically repeated population-based studies of serum lipid distributions are needed to determine physiological fluctuations in serum lipid concentrations among children and adolescents according to sex, chronological age and pubertal stage as well as changes in trends of serum lipid and lipoprotein distribution over time.

## Supplementary information

**Additional file 1.** LOESS curves with automatic smoothing parameter selection. The Additional file 1 presents the distribution of serum lipids stratified by sex and pubertal stage provided with automatic smoothing parameter selection.

**Additional file 2.** Description of LOESS methods. The Additional file 2 provides a brief description of LOESS methods.

**Additional file 3 **. Sensitivity analysis immigration background. The Additional file 3 contains an additional table (**Table S1**) presenting results of linear regression models of serum cholesterol parameters on pubertal stage among boys and girls adjusted for chronological age and immigration background.

## Data Availability

The authors confirm that some access restrictions apply to the data underlying the findings. The data set cannot be made publicly available because informed consent from study participants did not cover public deposition of data. However, the minimal data set underlying the findings is archived in the ‘Health Monitoring’ Research Data Centre at the Robert Koch Institute (RKI) and can be accessed by all interested researchers. On-site access to the data set is possible at the Secure Data Center of the RKI’s ‘Health Monitoring’ Research Data Centre. Requests should be submitted to the ‘Health Monitoring’ Research Data Centre, Robert Koch Institute, Berlin, Germany (e-mail: fdz@rki.de).

## Abbreviations

AAP: American Academy of Pediatrics
BMI: Body mass index
HDL-C: High-density lipoprotein cholesterol
HFD: Healthy Food Diversity Index
KiGGS: German Health Interview and Examination Survey for Children and Adolescents
NHLBI: National Heart, Lung, and Blood Institute
Non-HDL-C: *Non*-high-*density* lipoprotein cholesterol
TC: Total cholesterol

## References

[1] Expert Panel on Blood Cholesterol Levels in Children and Adolescents: National Cholesterol Education Program (NCEP): highlights of the report of the Expert Panel on Blood Cholesterol Levels in Children and Adolescents. Pediatrics. 1992;89(3):495–501. 1741227

[2] McMahan CA, Gidding SS, Malcom GT, Tracy RE, Strong JP, McGill HC (2006). Pathobiological determinants of atherosclerosis in youth risk scores are associated with early and advanced atherosclerosis. Pediatrics.

[3] Raitakari OT, Juonala M, Kahonen M, Taittonen L, Laitinen T, Maki-Torkko N (2003). Cardiovascular risk factors in childhood and carotid artery intima-media thickness in adulthood: the cardiovascular risk in young Finns study. JAMA.

[4] Vinci SR, Rifas-Shiman SL, Cheng JK, Mannix RC, Gillman MW, de Ferranti SD (2014). Cholesterol testing among children and adolescents during health visits. JAMA.

[5] American Academy of Pediatrics. Committee on Nutrition (1998). American Academy of Pediatrics. Committee on Nutrition. Cholesterol in childhood. Pediatrics.

[6] Kavey Rae-Ellen W, Daniels Stephen R, Lauer Ronald M, Atkins Dianne L, Hayman Laura L, Taubert K (2003). American Heart Association guidelines for primary prevention of atherosclerotic cardiovascular disease beginning in childhood. Circulation.

[7] Expert Panel on Integrated Guidelines for Cardiovascular Health and Risk Reduction in Children and Adolescents (2011). National Heart Lung and Blood Institute. Expert panel on integrated guidelines for cardiovascular health and risk reduction in children and adolescents: summary report. Pediatrics.

[8] American Academy of Pediatrics (2017). Bright Futures/AAP recommendations for preventive pediatric health care (periodicity schedule).

[9] U. S. Preventive Services Task Force (2016). Screening for lipid disorders in children and adolescents: us preventive services task force recommendation statement. JAMA.

[10] Working Group for pediatric metabolic disorders (APS) of the German Society for Pediatric and Adolescent Medicine (DGKJ) [S2k-Guideline 027/068. Diagnostics and therapy of hyperlipidaemia in children and adolescents] 2015. Available from: https://www.awmf.org/uploads/tx_szleitlinien/027-068l_s2k_Hyperlipid%C3%A4mien_Kinder_Jugendliche_2016-02.pdf. Accessed 4 Mar 2019.

[11] Wiegman A, Gidding SS, Watts GF, Chapman MJ, Ginsberg HN, Cuchel M (2015). Familial hypercholesterolaemia in children and adolescents: gaining decades of life by optimizing detection and treatment. Eur Heart J.

[12] Kordonouri O, Lange K, Boettcher I, Christoph J, Marquardt E, Tombois C (2019). New approach for detection of LDL-hypercholesterolemia in the pediatric population: the Fr1dolin-trial in Lower Saxony, Germany. Atherosclerosis.

[13] Lozano P, Henrikson N, Dunn J, Morrison C, Nguyen M, Whitlock E (2016). Lipid screening in childhood for detection of multifactorial dyslipidemia: a systematic evidence review for the U.S. preventive services task force. Evidence synthesis no. 140. AHRQ publication No14–05204-EF-1.

[14] Ford ES, Li C, Zhao G, Mokdad AH (2009). Concentrations of low-density lipoprotein cholesterol and total cholesterol among children and adolescents in the United States. Circulation.

[15] Hickman TB, Briefel RR, Carroll MD, Rifkind BM, Cleeman JI, Maurer KR (1998). Distributions and trends of serum lipid levels among United States children and adolescents ages 4-19 years: data from the third national health and nutrition examination survey. Prev Med.

[16] Jolliffe CJ, Janssen I (2006). Distribution of lipoproteins by age and gender in adolescents. Circulation.

[17] Twisk JW, Kemper HC, Mellenbergh GJ (1995). Longitudinal development of lipoprotein levels in males and females aged 12-28 years: the Amsterdam growth and health study. Int J Epidemiol.

[18] Eissa MA, Mihalopoulos NL, Holubkov R, Dai S, Labarthe DR (2016). Changes in fasting lipids during puberty. J Pediatr.

[19] Morrison JA, Laskarzewski PM, Rauh JL, Brookman R, Mellies M, Frazer M (1979). Lipids, lipoproteins, and sexual maturation during adolescence: the Princeton maturation study. Metab Clin Exp.

[20] Bertrais S, Balkau B, Charles MA, Vol S, Calvet C, Tichet J (2000). Puberty-associated differences in total cholesterol and triglyceride levels according to sex in French children aged 10-13 years. Ann Epidemiol.

[21] Tell GS, Mittelmark MB, Vellar OD (1985). Cholesterol, high density lipoprotein cholesterol and triglycerides during puberty: the Oslo youth study. Am J Epidemiol.

[22] Kleiser C, Schienkiewitz A, Schaffrath Rosario A, Prinz-Langenohl R, Scheidt-Nave C, Mensink GB (2011). Indicators of overweight and cardiovascular disease risk factors among 11- to 17-year-old boys and girls in Germany. Obes Facts.

[23] Enkhmaa B, Surampudi P, Anuurad E, Berglund L (2000). Lifestyle Changes: Effect of Diet, Exercise, Functional Food, and Obesity Treatment, on Lipids and Lipoproteins. [Updated 2015 Jun 8].

[24] Holmes L, LaHurd A, Wasson E, McClarin L, Dabney K (2015). Racial and Ethnic Heterogeneity in the Association Between Total Cholesterol and Pediatric Obesity. Int J Environ Res Public Health.

[25] Frank AT, Zhao B, Jose PO, Azar KM, Fortmann SP, Palaniappan LP (2014). Racial/ethnic differences in dyslipidemia patterns. Circulation.

[26] Kurth BM, Kamtsiuris P, Holling H, Schlaud M, Dolle R, Ellert U (2008). The challenge of comprehensively mapping children's health in a nation-wide health survey: design of the German KiGGS-study. BMC Public Health.

[27] Schaffrath-Rosario A, Kurth BM, Stolzenberg H, Ellert U, Neuhauser H (2010). Body mass index percentiles for children and adolescents in Germany based on a nationally representative sample (KiGGS 2003-2006). Eur J Clin Nutr.

[28] Cole TJ, Bellizzi MC, Flegal KM, Dietz WH (2000). Establishing a standard definition for child overweight and obesity worldwide: international survey. BMJ (Clinical research ed).

[29] Lampert T (2008). Smoking and passive smoking exposure in young people: results of the German health interview and examination survey for children and adolescents (KiGGS). Dtsch Arztebl Int.

[30] Lampert T, Mensink GB, Romahn N, Woll A (2007). Physical activity among children and adolescents in Germany. Results of the German health interview and examination survey for children and adolescents (KiGGS). Bundesgesundheitsblatt Gesundheitsforschung Gesundheitsschutz.

[31] Truthmann J, Richter A, Thiele S, Drescher L, Roosen J, Mensink GB (2012). Associations of dietary indices with biomarkers of dietary exposure and cardiovascular status among adolescents in Germany. Nutr Metabo.

[32] Hintzpeter B, Scheidt-Nave C, Müller MJ, Schenk L, Mensink GBM (2008). Higher prevalence of vitamin D deficiency is associated with immigrant background among children and adolescents in Germany. J Nutr.

[33] Marshall WA, Tanner JM (1970). Variations in the pattern of pubertal changes in boys. Arch Dis Child.

[34] Marshall WA, Tanner JM (1969). Variations in pattern of pubertal changes in girls. Arch Dis Child.

[35] Finne E, Bucksch J, Lampert T, Kolip P (2011). Age, puberty, body dissatisfaction, and physical activity decline in adolescents. Results of the German Health Interview and Examination Survey (KiGGS). Int J Behav Nutr Phys Act.

[36] Holling H, Kamtsiuris P, Lange M, Thierfelder W, Thamm M, Schlack R (2007). The German health interview and examination survey for children and adolescents (KiGGS): study management and conduct of fieldwork. Bundesgesundheitsblatt Gesundheitsforschung Gesundheitsschutz.

[37] Thierfelder W, Dortschy R, Hintzpeter B, Kahl H, Scheidt-Nave C (2007). Biochemical measures in the German health interview and examination survey for children and adolescents (KiGGS). Bundesgesundheitsblatt Gesundheitsforschung Gesundheitsschutz..

[38] Agirbasli M, Agaoglu NB, Orak N, Caglioz H, Ocek T, Karabağ T (2010). Sex hormones, insulin resistance and high-density lipoprotein cholesterol levels in children. Hormone Res Paediatr.

[39] Papadimitriou A, Chrousos GP (2005). Reconsidering the sex differences in the incidence of pubertal disorders. Horm Metab Res = Hormon- und Stoffwechselforschung = Hormones et Metabolisme.

[40] Ferrández A, Carrascosa A, Audí L, Baguer L, Rueda C, Bosch-Castañé J, et al. Longitudinal Pubertal Growth According to Age at Pubertal Growth Spurt Onset: Data from a Spanish Study Including 458 Children (223 Boys and 235 Girls). J Pediatr Endocrinol Metab. 2009;22(8):715–26. 10.1515/jpem.2009.22.8.715 19845122

[41] Mellerio H, Alberti C, Druet C, Capelier F, Mercat I, Josserand E (2012). Novel modeling of reference values of cardiovascular risk factors in children aged 7 to 20 years. Pediatrics.

[42] Hardy R, Langenberg C (2003). Commentary: the association between height growth and cholesterol levels during puberty: implications for adult health. Int J Epidemiol.

[43] Dobbins M, DeCorby K, Robeson P, Husson H, Tirilis D. School-based physical activity programs for promoting physical activity and fitness in children and adolescents aged 6-18. Cochrane Database Syst Rev. Cochrane Database Syst Rev. 2009. 10.1002/14651858.CD007651. 10.1002/14651858.CD007651 19160341

[44] Temple NJ (2018). Fat, sugar, whole grains and heart disease: 50 years of confusion. Nutrients.

[45] Steiner MJ, Skinner AC, Perrin EM (2011). Fasting might not be necessary before lipid screening: a nationally representative cross-sectional study. Pediatrics.

[46] Schwab KO, Doerfer J, Naeke A, Rohrer T, Wiemann D, Marg W (2009). Influence of food intake, age, gender, HbA1c, and BMI levels on plasma cholesterol in 29,979 children and adolescents with type 1 diabetes--reference data from the German diabetes documentation and quality management system (DPV). Pediatr Diabetes.

[47] Rasmussen AR, Wohlfahrt-Veje C, Tefre de Renzy-Martin K, Hagen CP, Tinggaard J, Mouritsen A (2015). Validity of self-assessment of pubertal maturation. Pediatrics.

[48] Ernst Andreas, Lauridsen Lea Lykke B., Brix Nis, Kjersgaard Camilla, Olsen Jørn, Parner Erik T., Clausen Niels, Olsen Lars Henning, Ramlau-Hansen Cecilia H. (2018). Self-assessment of pubertal development in a puberty cohort. Journal of Pediatric Endocrinology and Metabolism.

[49] Biro FM, Greenspan LC, Galvez MP (2012). Puberty in girls of the 21st century. J Pediatr Adolesc Gynecol.

[50] Herman-Giddens ME, Steffes J, Harris D, Slora E, Hussey M, Dowshen SA (2012). Secondary sexual characteristics in boys: data from the pediatric research in office settings network. Pediatrics.

[51] Toppari J, Juul A (2010). Trends in puberty timing in humans and environmental modifiers. Mol Cell Endocrinol.

[52] Cobbaert C, Deprost L, Mulder P, Rombaut K, Gijsels G, Kesteloot H (1995). Pubertal serum lipoprotein (a) and its correlates in Belgian schoolchildren. Int J Epidemiol.

[53] Lauer RM, Lee J, Clarke WR (1988). Factors affecting the relationship between childhood and adult cholesterol levels: the Muscatine study. Pediatrics.

[54] Freedman DS, Wang YC, Dietz WH, Xu JH, Srinivasan SR, Berenson GS (2010). Changes and variability in high levels of low-density lipoprotein cholesterol among children. Pediatrics.

[55] Perak AM, Ning H, Kit BK, de Ferranti SD, Van Horn LV, Wilkins JT (2019). Trends in levels of lipids and Apolipoprotein B in US youths aged 6 to 19 years, 1999-2016. JAMA.

