# Supplementary material for: Age, maturation and serum lipid parameters: findings from the German Health Survey for Children and Adolescents
Source: BMC Public Health. 2019 Dec 3;19:1627. doi: 10.1186/s12889-019-7901-z (PMC6891966; doi:10.1186/s12889-019-7901-z)
Supplement: Supplementary file 1 — Additional file 1. LOESS curves with automatic smoothing parameter selection. The Additional file 1 presents the distribution of serum lipids stratified by sex and pubertal stage provided with automatic smoothing parameter selection. [file 12889_2019_7901_MOESM1_ESM.docx]

**Additional File 1: LOESS curves with automatic smoothing parameter selection**

**Figure S1: Distribution of serum lipids stratified by sex and pubertal stage provided with automatic smoothing parameter selection**

|  | Boys | Girls |
| --- | --- | --- |
| TC* | 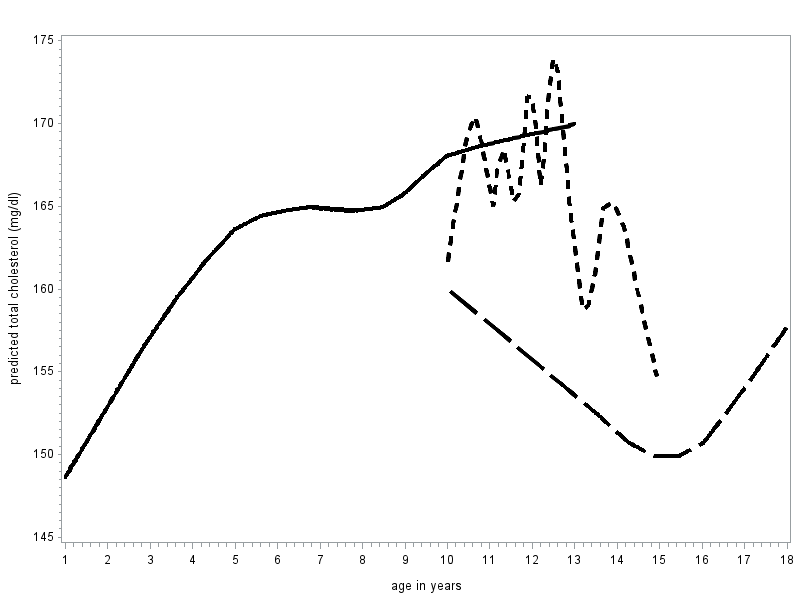 | 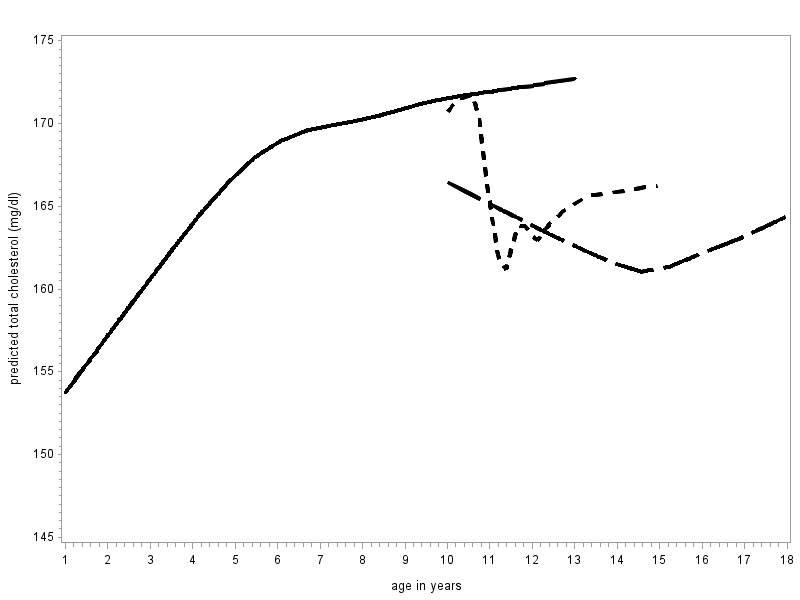 |
| HDL* | 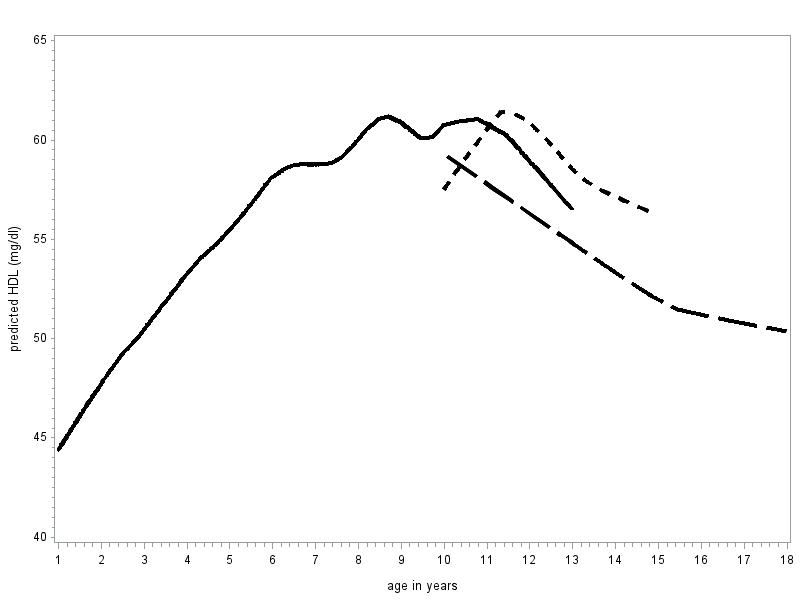 | 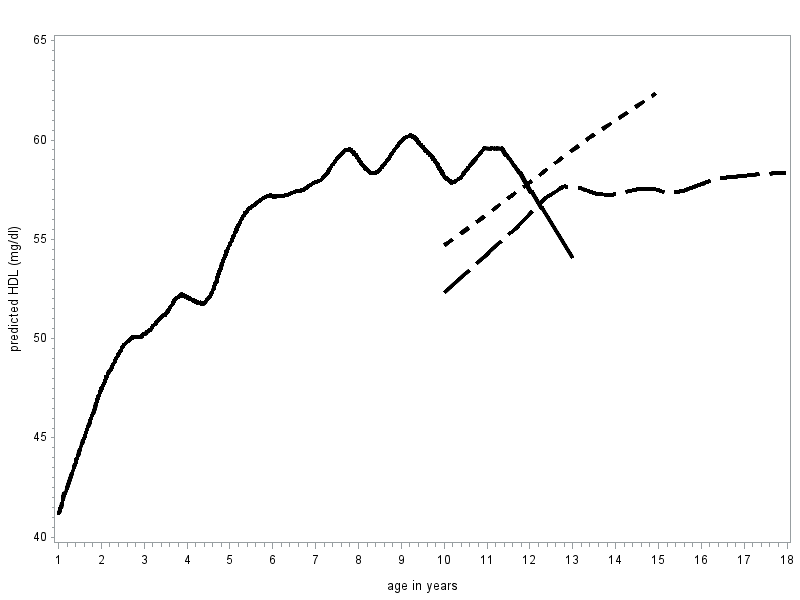 |
| Non-HDL* | 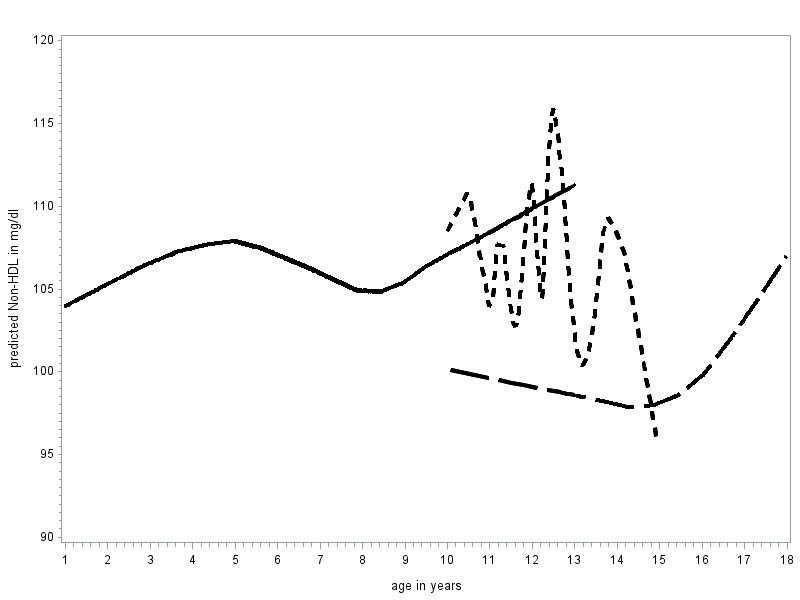 | 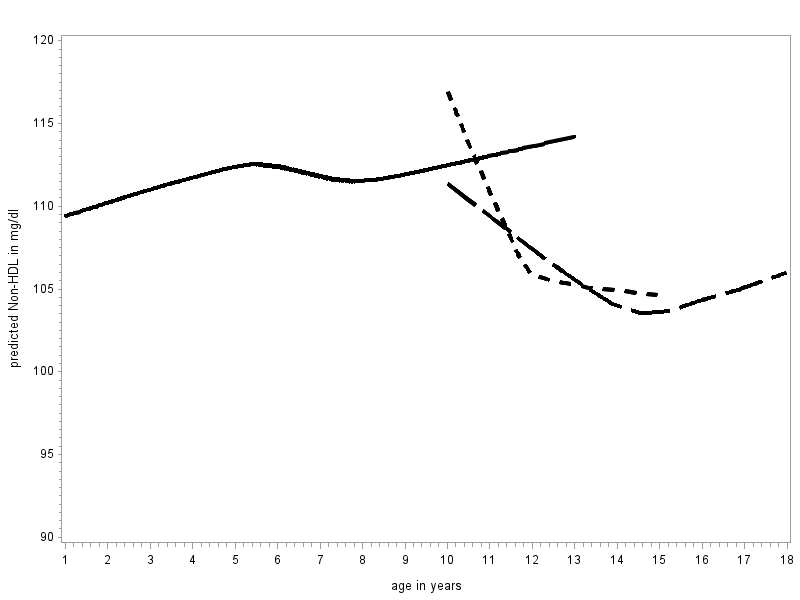 |

* solid line = prepubescent, short broken line = early/mid-puberty, long broken line = mature/advanced puberty
