# Supplementary material for: Age, maturation and serum lipid parameters: findings from the German Health Survey for Children and Adolescents
Source: BMC Public Health. 2019 Dec 3;19:1627. doi: 10.1186/s12889-019-7901-z (PMC6891966; doi:10.1186/s12889-019-7901-z)
Supplement: Supplementary file 2 — Additional file 2. Description of LOESS methods. The Additional file 2 provides a brief description of LOESS methods. [file 12889_2019_7901_MOESM2_ESM.docx]

**Additional File 2: Description of LOESS methods**

Locally weighted regression models estimate a regression surface through a multivariate smoothing procedure, fitting a function of the independent variables locally, i.e. for each of the data points whereas nearer neighbors have a greater weight than neighbors further away. A smoothing parameter can be specified to determine a fraction of data points which will be used for fitting the function. With local fitting a much wider class of regression surfaces than with the usual classes of parametric functions, such as polynomials can be estimated. In addition, the impact of extreme values is smaller than in parametric regression models^1^.

The LOESS procedure was applied to different subgroups according to sex and pubertal status. The LOESS procedure provides automatic smoothing parameter selection using bias-corrected AIC criteria, and generalized cross-validation (http://support.sas.com/rnd/app/da/new/801ce/stat/chap6/sect7.htm). However some of the curves showed strong fluctuations which are not plausible. In that case higher smoothing parameters where chosen by visual inspection.

^1^Cleveland WS, Devlin ST. Locally Weighted Regression: An Approach to Regression Analysis by Local Fitting. *Journal of the American Statistical Association.* 1988;83(403):596-610.
