# Supplementary material for: Age, maturation and serum lipid parameters: findings from the German Health Survey for Children and Adolescents
Source: BMC Public Health. 2019 Dec 3;19:1627. doi: 10.1186/s12889-019-7901-z (PMC6891966; doi:10.1186/s12889-019-7901-z)
Supplement: Supplementary file 3 — Additional file 3 . Sensitivity analysis immigration background. The Additional file 3 contains an additional table (Table S1) presenting results of linear regression models of serum cholesterol parameters on pubertal stage among boys and girls adjusted for chronological age and immigration background. [file 12889_2019_7901_MOESM3_ESM.docx]

**Additional File 3: Sensitivity analysis immigration background**

**Table S1: Linear regression of serum cholesterol parameters on pubertal stage among boys and girls adjusted for age and immigration background**

|  |  | **TC [mg/dL]** | **HDL-C [mg/dL]** | **Non-HDL-C [mg/dL]** |
| --- | --- | --- | --- | --- |
|  |  |  |  |  |
| **Boys (n=7164)** |  |  |  |  |
| **Model 1: unadjusted** | R^2^ | 0.04 | 0.04 | 0.01 |
| **Early/mid-puberty** | β (S.E.) | 3.7 (1.2)** | 3.2 (0.5)*** | 0.4 (1.1) |
| **Mature/advanced puberty** | β (S.E.) | -10.6 (0.9)*** | -4.4 (0.4)*** | -6.2 (0.8)*** |
| **Model 2: adj. for age** | R^2^ | 0.05 | 0.11 | 0.01 |
| **Early/mid-puberty** | β (S.E.) | -3.0 (1.4)* | -1.5 (0.7)* | -1.5 (1.3) |
| **Mature/advanced puberty** | β (S.E.) | -17.5 (1.8)*** | -6.4 (0.9)*** | -11.1 (1.7)*** |
| **Model 3: adj. for age and immigration background** | R^2^ | 0.05 | 0.11 | 0.01 |
| **Early/mid-puberty** | β (S.E.) | -3.0 (1.4)* | -1.5 (0.7)* | -1.5 (1.3) |
| **Mature/advanced puberty** | β (S.E.) | -17.5 (1.8)*** | -6.4 (0.9)*** | -11.1 (1.7)*** |
| **Immigration background (yes vs. no)** | β (S.E.) | 0.1 (1.1) | -0.1 (0.5) | 0.2 (1.0) |
|  |  |  |  |  |
| **Girls (n=6453**) |  |  |  |  |
| **Model 1: unadjusted** | R^2^ | 0.01 | 0.01 | 0.01 |
| **Early/mid-puberty** | β (S.E.) | -1.3 (1.4) | 2.6 (0.7)** | -3.9 (1.4)** |
| **Mature/advanced puberty** | β (S.E.) | -4.7 (0.9)*** | 2.4 (0.4)*** | -7.1 (0.8)*** |
| **Model 2: adj. for age** | R^2^ | 0.02 | 0.08 | 0.01 |
| **Early/mid-puberty** | β (S.E.) | -7.7 (1.7)*** | -4.1 (0.8)*** | -3.6 (1.7)* |
| **Mature/advanced puberty** | β (S.E.) | -10.7 (2.0)*** | -5.0 (0.8)*** | -5.7 (1.9)* |
| **Model 3: adj. for age and immigration background** | R^2^ | 0.02 | 0.08 | 0.02 |
| **Early/mid-puberty** | β (S.E.) | -7.7 (1.7)*** | -4.1 (0.8)*** | -3.7 (1.7)* |
| **Mature/advanced puberty** | β (S.E.) | -10.7 (2.0)*** | -5.0 (0.8)*** | -5.7 (1.9)* |
| **Immigration background (yes vs. no)** | β (S.E.) | -1.3 (1.2) | 0.1 (0.5) | -1.4 (1.2) |

β = Regression coefficient, S.E. = Standard error, R^2^ = proportion of variance explained by the model, Reference = prepubescent stage

* p<0.05, ** p<0.001, *** p<0.0001
